# COVID-19 in Pediatric Granulomatosis with Polyangiitis

**DOI:** 10.3390/pediatric13010004

**Published:** 2021-01-04

**Authors:** Amir Saeed, Eslam Shorafa, Zahra Seratishirazi, Seyedenarjes Abootalebi

**Affiliations:** Department of Pediatrics, Division of Intensive Care Unit, Shiraz University of Medical Sciences, Shiraz 71348, Iran; dr.saeedamir@yahoo.com (A.S.); zserati@yahoo.co.uk (Z.S.); abootalebinarjes@yahoo.com (S.A.)

**Keywords:** COVID19, coronavirus, wegener, granulomatosis with polyangiitis

## Abstract

The confirmed cases with COVID-19 in children account for just 1% of the overall confirmed cases. Severe COVID-19 in children is rare. Case Presentation: Our patient was 16 years old with a severe case of COVID-19 and did not survive due to the presence of Granulomatosis with polyangiitis and being treated with immunosuppressive drugs. We used lopinavir, ritonavir, hydroxy chloroquine, intravenous immunoglobulin and continuous veno-venous hemodialysis for treatment. Conclusion: In this patient, an underlying disease and delayed admission to the hospital were two factors complicating his condition.

## 1. Introduction

The outbreak of COVID-19 started as an epidemic in Wuhan, China and caught world-wide attention as a pandemic in January 2020. Confirmed pediatric cases account for just 1% of total cases [1].

Severe COVID-19 infections in children are rare. To date, the largest review of children with COVID-19 included 2143 children in China. Only 112 (5.6%) of 2143 children infected presented with severe symptoms (defined as hypoxia) and 13 (0.6%) children developed respiratory distress syndrome (ARDS) or multi-organ failure [2].

Our patient had a severe case of COVID-19 and did not survive due to the presence of Granulomatosis with polyangiitis and being treated with immunosuppressive drugs.

Rheumatological diseases and inflammatory bowel disease are immune disorders that are associated with an increased risk of opportunistic and community-acquired infections such as respiratory virus infections. They are at risk of high mortality and co-morbidity [3].

For patients with rheumatic disease, there is little data to understand the real consequences of the infection. Pediatric rheumatologists are expected to play a supporting role in treatment of COVID-19, both as pediatricians treating infected children, and as rheumatologists taking care of their rheumatic patients, as well as offering their experience in the possible alternative use of immunomodulatory drugs [4].

## 2. Case Presentation

A 16 year-old, 80-kg patient was referred to a tertiary hospital in Shiraz, Iran on 27 March 2020. He had a known case of Granulomatosis with polyangiitis and was diagnosed 2 years prior. At that time the patient presented with headache, sore throat, conjunctivitis for 1 month that was followed by lower extremities edema and joints pain and skin rash, and the Granulomatosis with polyangiitis was distinguished. His condition was in remission according to the Birmingham Vasculitis Activity Score for Wegener’s Granulomatosis Evaluation Form [5] before recent disease. The patient was on these medications before admission: prednisolone 10 milligrams (mg) daily, mycophenolate 360 mg twice per day, aspirin 80 mg daily, valsartan 10 mg daily, allopurinol 100 mg daily and folic acid. The patient’s complaint started with coughing and rhinorrhea 7 days prior to admission. His condition worsened over the following few days. He had very limited activity in the 3 days prior to admission. He displayed shortness of breath and preferred to lie down in a prone position.

On the day of admission, he was intubated in the emergency room due to respiratory distress and decreased O_2_ Saturation (O_2_ Sat) of 70%. He was transferred to the pediatric intensive care unit (PICU). In the PICU, a ventilator was set up on SIMV with a respiratory rate of 20/min, tidal volume of 500 mL, inspiratory time: 1min, peak end expiratory pressure (PEEP): 15 cm H_2_O and FIO_2_: 100%. An infusion of norepinephrine was administered due to hypotension. Covid-19 RT-PCR came back positive. There was no history of exposure to confirmed or suspected coronavirus cases.

His medication continued except valsartan. Prednisolone changed to stress dose of hydrocortisone.

Lopinavir, ritonavir, hydroxy chloroquine, vancomycin and meropenem started. He also received intravenous immunoglobulin (IVIG) the first day of admission because of his refractory shock.

**Figure 1:**
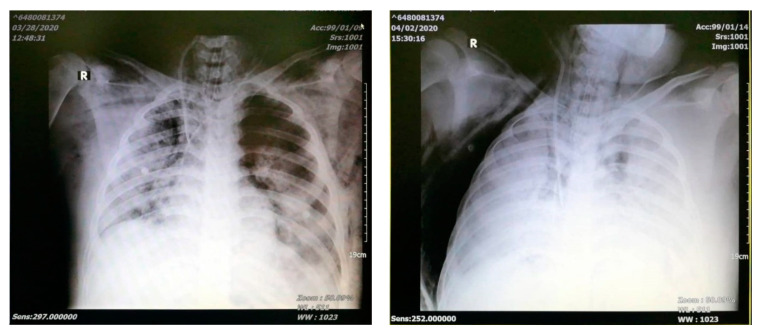
Chest X-ray on day 1 (**left**) and day 4 (**right**).

Chest X-ray on admission showed bilateral patchy infiltration which deteriorated to white lung on day 6 (Figure 1). A CT scan was not done due to his severe condition and the lack of a portable ventilator with high PEEP set up. All lab tests are shown in Table 1. 

On day 3 he had atrial fibrillation with rapid ventricular response. After emergency intervention, flecainide began with pediatric cardiologist consultation.

He also became anuric on day 4 and continuous veno-venous hemodialysis started for him. His condition deteriorated over time with a decrease in O_2_ Sat in spite of 100% of FIO_2_ and an increase in PEEP to 22cm H_2_O. On day 3, he was put into a prone positioning which helped to increase O_2_ Sat (up to 97%). However, it started to decline again after 36 h.

Unfortunately, the patient expired on day 6 of admission with low O_2_ Sat and bradycardia.

## 3. Discussion

COVID-19 mortality in children is very rare. The data from a low number of children suggest that even children who are on immunosuppressive treatment for various indications have a mild clinical course of COVID-19. Additionally, a study with eight children with inflammatory bowel disease (IBS), despite treatment with immunomodulators, biologics, or both, shows that all children diagnosed with COVID-19 had a mild infection [6].

But in this patient, an underlying disease and delayed admission to the hospital were two factors complicating his condition.

In addition, while there was extensive publicity for home quarantine, it is important to note that fears of contracting coronavirus may cause people to delay going to health centers until it is too late to treat them effectively. Some patients, concerned about over-burdening the healthcare system, may also delay seeking hospital treatment until their symptoms become critical.

The existing data on past and present coronavirus outbreaks show that immunosuppressed patients are not at increased risk of severe pulmonary disease compared with the general population. Children under the age of 12 years do not progress to severe coronavirus pneumonia, without paying attention to their immune status, although they get infected and can spread the infection [7].

Screening those who care for these high-risk patients is also suggested.

## Figures and Tables

**Table 1:** Lab tests from day 1 to day 6 of admission.

Day of Admission →	1	2	3	4	5	6
Lab Data ↓						
COVID RT-PCR	Positive					
Influenza RT-PCR	Negative					
Blood urea nitrogen (mg/dl)	53	59	67	68	87	96
creatinine	4.8	5.5	5.2	5.9	7.9	8.8
Sodium (mEq/L)	130	135	139	134	135	136
Potassium (mEq/L)	6	5.4	3.7	4.5	5.6	5.2
Calcium (mg/dL)	8.3		8.9		8.4	
Troponin (ng/mL)	450	1688				
D.Dimer (ng/mL)		650		7627		
Lactate dehydrogenase (U/L)	1350	1340				
Creatine phosphokinase (U/L)		119	137	537	1030	1040
Magnesium (mg/dL)	2.4		1.9		3.1	
Ferritin (ng/mL)			1310			
Procalcitonin (ng/mL)	5.77					
Aspartate transaminase (U/L)		43				
Alanine aminotransferase (U/L)		13				
White blood cells(count/mL)	22,800	14,800	17,200	22,900	15,800	16,200
Hemoglobin (g/dL)	9.6	7.8	11.1 *	10.5	9	8
Platelet (count/mL)	494,000	338,000	339,000	325,000	234,000	196,000
Prothrombin time	12	12.1	13.6	18.1+	20+	15.3
Partial thromboplastin time	25	24	33	41	47	38
INR	1	1.01	1.2	1.8	2.13	1.4

HIV antibody, HCV antibody, HBS antigen were negative. TB work up was negative. * red blood cell transfusion, +fresh frozen plasma transfusion.

## Data Availability

All data relevant to the study are included in the article or uploaded as supplementary information.
